# Molecular pathophysiology of secondary lymphedema

**DOI:** 10.3389/fcell.2024.1363811

**Published:** 2024-07-08

**Authors:** Sang-Oh Lee, Il-Kug Kim

**Affiliations:** Department of Plastic and Reconstructive Surgery, College of Medicine, Yeungnam University, Daegu, Republic of Korea

**Keywords:** lymphedema, lymphatic system, physiopathology, molecular biology, inflammation, fibrosis, adipose tissue

## Abstract

Lymphedema occurs as a result of lymphatic vessel damage or obstruction, leading to the lymphatic fluid stasis, which triggers inflammation, tissue fibrosis, and adipose tissue deposition with adipocyte hypertrophy. The treatment of lymphedema is divided into conservative and surgical approaches. Among surgical treatments, methods like lymphaticovenular anastomosis and vascularized lymph node transfer are gaining attention as they focus on restoring lymphatic flow, constituting a physiologic treatment approach. Lymphatic endothelial cells form the structure of lymphatic vessels. These cells possess button-like junctions that facilitate the influx of fluid and leukocytes. Approximately 10% of interstitial fluid is connected to venous return through lymphatic capillaries. Damage to lymphatic vessels leads to lymphatic fluid stasis, resulting in the clinical condition of lymphedema through three mechanisms: Inflammation involving CD4^+^ T cells as the principal contributing factor, along with the effects of immune cells on the VEGF-C/VEGFR axis, consequently resulting in abnormal lymphangiogenesis; adipocyte hypertrophy and adipose tissue deposition regulated by the interaction of CCAAT/enhancer-binding protein α and peroxisome proliferator-activated receptor-γ; and tissue fibrosis initiated by the overactivity of Th2 cells, leading to the secretion of profibrotic cytokines such as IL-4, IL-13, and the growth factor TGF-β1. Surgical treatments aimed at reconstructing the lymphatic system help facilitate lymphatic fluid drainage, but their effectiveness in treating already damaged lymphatic vessels is limited. Therefore, reviewing the pathophysiology and molecular mechanisms of lymphedema is crucial to complement surgical treatments and explore novel therapeutic approaches.

## Introduction

Lymphedema is a condition characterized by the accumulation of lymphatic fluid due to the obstruction or destruction of lymphatic vessels. This leads to progressive fibrosis, adipocyte hypertrophy and adipose tissue deposition. It is classified into two main types: primary lymphedema, which is caused by genetic or developmental abnormalities, and secondary lymphedema, which is triggered by external factors such as trauma, radiation therapy, recurrent infections, cancer surgery, obesity, and other causes (Zampell et al., 2012a; Duhon et al., 2022; Sleigh and Manna, 2024). Secondary lymphedema arises due to injury or obstruction of the lymphatic system, and globally, the most common cause is attributed to filariasis, a condition in which parasitic worms invade lymphatic vessels, leading to their blockage (Douglass et al., 2016). In developed countries, the most significant contributing factor to secondary lymphedema is often complications arising after cancer treatments such as surgery, radiation therapy, and chemotherapy (Brown et al., 2023a).

Lymphatic capillaries possess a discontinuous basement membrane, facilitating the influx of immune cells, cell debris, proteins, and other substances. When injury occurs to the lymphatic system, the inflow of interstitial fluid into lymphatic capillaries becomes impaired, leading to lymphatic fluid stasis. This condition can result in significant processes such as inflammation, fibrosis, and deposition of adipose tissue (Null et al., 2024).

Early-stage lymphedema can often be cured with non-surgical treatments, while advanced-stage lymphedema typically requires surgical intervention for management. However, there is currently no clinically proven effective drug therapy available. Recently, surgical procedures aimed at restoring lymphatic circulation, such as lymphaticovenular anastomosis and vascularized lymph node transfer, using super-microsurgery techniques, have been performed in lymphedema patients. Nevertheless, these physiologic procedures have their limitations in terms of surgical outcomes, necessitating the development of new treatment modalities to complement them.

The main objective of this paper is to thoroughly analyze the complex pathophysiology and molecular mechanisms of lymphedema in order to explore strategies for complementing and improving current treatment methods. By reviewing current conservative and surgical treatment approaches, it is pointed out that while the latest surgical treatments can restore lymphatic drainage function, their effectiveness in treating already damaged lymphatic vessels may be limited. Based on this, the emphasis of this review lies in the pursuit of developing more effective treatment strategies by gaining a deeper understanding of the pathological processes.

### Structure and function of lymphatic vessels

Lymphatic capillaries differentiate from venous endothelial cells. After arteriovenous differentiation is regulated by Notch and chicken ovalbumin upstream promoter transcription factor II (COUP-TFII), SRY-box 18 (SOX 18) activates prospero homeobox-1 (Prox-1), which interacts with COUP-TFII to promote the differentiation into lymphatic endothelial cells (LECs) and increase the expression of VEGFR-3 (François et al., 2008; Srinivasan et al., 2010). It functions as a receptor tyrosine kinase for the lymphangiogenic growth factors VEGF-C and VEGF-D (Mäkinen et al., 2001). Lymph sacs develop while maintaining a connection to adjacent veins, a critical site where lymphovenous valves form and interstitial fluid is collected into blood circulation (Knowlton, 1970; van der Putte, 1975). VEGFR-3 plays a pivotal role in this process through an autoregulatory feedback mechanism that regulates Prox-1, essential for maintaining the specification and identity of Prox-1+ LEC progenitors in the cardinal vein (Wigle and Oliver, 1999; Oliver, 2004; Srinivasan et al., 2014). Subsequently, VEGF-C undergoes proteolytic processing by calcium-binding EGF domain-1 protein (Ccbe1) and metalloproteinase with thrombospondin motifs 3 (Adamts3), binding to VEGFR-3 and inducing the sprouting of initial lymphatic vessels from the cardinal vein (Karkkainen et al., 2004; Hogan et al., 2009; Bui et al., 2016). During this process, the co-receptor neuropilin-2 (NRP-2) binds to VEGF-C, while the Eph tyrosine kinase ligand ephrin-B2 promotes VEGFR-3 internalization, and β1-integrin, responding to increased interstitial fluid, facilitates VEGFR-3 phosphorylation, contributing to the sprouting of lymphatic capillaries (Wigle and Oliver, 1999; Yuan et al., 2002; Mäkinen et al., 2005; Srinivasan et al., 2010; Xu et al., 2010; Planas-Paz et al., 2012). In the process of lymphatic vessel maturation, the regulation of cell polarity and valve development is influenced by key proteins such as Celsr1, Vangl2, Pkd1, Pkd2, and FAT4. Moreover, the integrity of LEC junctions critical for controlling lumen size is managed by the Ras-interacting protein-1 (Rasip1) (Oliver et al., 2020).

Lymphatic vessel endothelial hyaluronan receptor-1 (LYVE-1) serves as a hyaluronan receptor in lymphatic capillaries but is less expressed in collecting vessels (Rinaldi and Baggi, 2018). Therefore, LYVE-1 expression can be used as a marker of lymphatic vessels; however, it is important to distinguish it from its presence in certain macrophages within various tissues (Karinen et al., 2022). The angiopoietins and Tie receptors also play roles in lymphatic sprouting and vessel defects (Augustin et al., 2009).

Lymphatic capillaries, characterized by their discontinuous basement membranes and lack of pericytes, feature button-like junctions, unlike blood capillaries that have zipper-like junctions (Baluk et al., 2007). These endothelial cells open in response to increased interstitial pressure, utilizing anchoring filaments to adjust ‘flap valve’ openings to facilitate the entry of various substances (Tammela and Alitalo, 2010; Alitalo, 2011; Zhang et al., 2020; Baluk and McDonald, 2022; Null et al., 2024). The lymphatic system plays crucial roles in draining interstitial fluid, fat absorption, and immune surveillance (Bittar et al., 2020), with about 90% of the fluid being reabsorbed into the venous system and the remaining high-protein fluid navigating through lymph nodes before reentering the bloodstream near the right atrium (Moore and Bertram, 2018).

**FIGURE 1:**
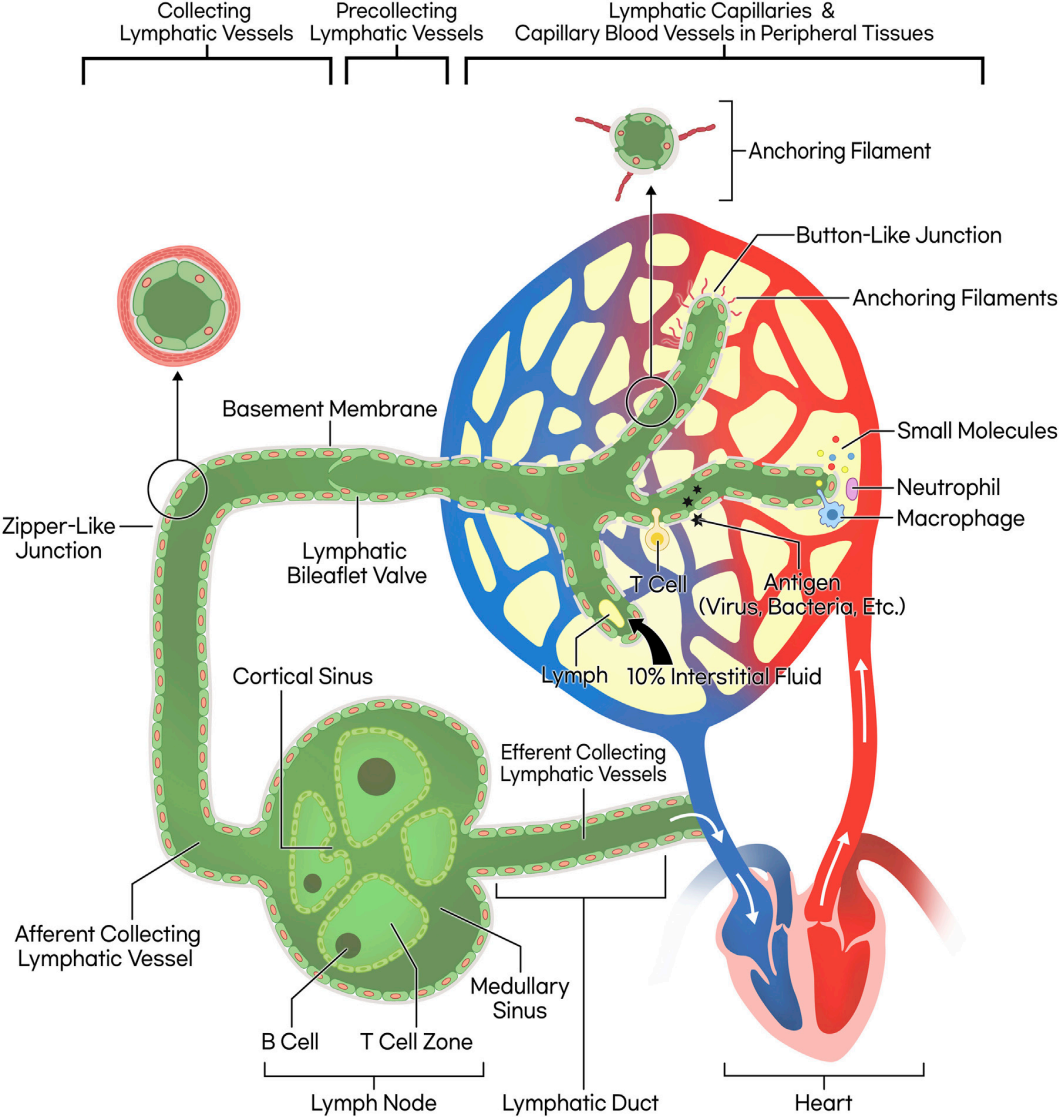
Schematic illustration of lymphatic circulation and lymphatic vessel structure. The lymphatic capillary possesses a discontinuous basement membrane and lacks pericytes that typically envelop blood endothelial cells, resulting in a button-like junction pattern. This structural characteristic facilitates the ingress of small molecules, fluid, and leukocytes. The collecting lymphatic vessel, on the other hand, features a smooth muscle layer, bileaflet valves, and zipper-like junctions, allowing lymph propulsion forward through wall contraction. Consequently, the lymph progresses through lymph nodes and lymphatic ducts, ultimately merging into venous return, completing systemic circulation.

Collecting lymphatic vessels, distinct from lymphatic capillaries, are equipped with smooth muscle layers, continuous zipper-like interendothelial junctions, and bileaflet valves to propel lymph forward and prevent backflow, in contrast to the more permeable lymphatic capillaries. These features ensure directional lymph flow and exclude fluid absorption from surrounding tissues (Norden and Kume, 2020; Null et al., 2024) (Figure 1.)

Fluid shear stress (FSS) affects lymphatic vessels by activating mechanotransduction in lymphatic endothelial cells (LECs), involving sensors like platelet endothelial cell adhesion molecule (PECAM), vascular endothelial (VE)-cadherin, VEGFR2, and VEGFR3. This activation triggers pathways such as phosphoinositide 3-kinases/protein kinase B (PI3K/Akt), leading to cytoskeleton reorganization and Yes-associated protein/transcriptional coactivator with PDZ-binding motif (YAP/TAZ) signaling, which responds to ECM stiffness and shear. PIEZO1, another mechanosensor, activates ORAI1, causing calcium influx that promotes valve formation via proteins like forkhead box C2 (FOXC2), connexin 37 (CX37), integrin alpha 9 (ITGA9), and GATA binding protein 2 (GATA2). Defects in FOXC2 result in abnormal vessel responses and hyperproliferation. (Geng et al., 2021; Geng and Srinivasan, 2022; Angeli and Lim, 2023). Additionally, FOXP2 and FAT4 regulate FSS-dependent LEC polarization, while TGF-β signaling, vital for proper lymphatic function, requires more research due to its complex roles in vessel expansion and fluid drainage (Betterman et al., 2020; Hernández Vásquez et al., 2021; Itoh and Watabe, 2022).

### Pathophysiology of secondary lymphedema: inflammation

In the pathophysiology of lymphedema, the inflammatory response involving CD4^+^ T cells is the most crucial mechanism, leading to the development of lymphedema (Li et al., 2020). In a mouse tail surgery and popliteal lymph node dissection (PLND) model, more than 70% of the inflammatory response was composed of CD4^+^ T cells, and when CD4^+^ T cells were depleted, the onset of lymphedema could be prevented. However, depletion of macrophages or CD8^+^ T cells did not have the same effect (García Nores et al., 2018). Research involving human specimens from unilateral upper extremity breast cancer-related lymphedema demonstrated that the number of CD4^+^ T cells was associated with the severity of lymphedema, and even a small number of these cells were sufficient to induce lymphedema (Zampell et al., 2012b; Avraham et al., 2013; Ly et al., 2019). These studies indicate that CD4^+^ T cells play a significant role in the development of lymphedema.

When mechanical interruptions occur in the lymphatic system, various growth factors and cytokines become upregulated, leading to lymphatic stasis. Among these growth factors, VEGF-C primarily activates VEGFR-3 to promote lymphangiogenesis and VEGFR-2 to enhance vascular permeability. This dual action is crucial for regenerating collateral lymphatic vessels. However, in cases of lymphatic system obstruction, elevated levels of VEGF-C can induce lymphatic hyperplasia, resulting in less efficient drainage and exacerbating lymphatic fluid stasis (Gousopoulos et al., 2017b). Due to fluid stasis, naive CD4^+^ T cells in the skin draining lymph nodes interact with antigen-presenting cells (APCs), leading to their activation. Subsequently, these activated CD4^+^ T cells infiltrate lymphedematous skin, promoting impaired lymphangiogenesis and fibrosis. Furthermore, they contribute to an increase in inducible nitric oxide synthase (iNOS), negatively affecting lymphatic pumping (Scallan and Davis, 2013; García Nores et al., 2018).

The CD4^+^ T cell infiltration process involves a mixed CD4^+^ T helper cell (Th) response, consisting of Th1, Th2, Th17, and T regulatory (Treg) cells (Kataru et al., 2019). Among these, Th2 cells are the most dominant, secreting TGF-β1, IL-4, and IL-13. These cytokines promote fibrosis through the differentiation of fibroblasts into myofibroblasts and production of extracellular matrix (ECM) products. Additionally, IL-4 and IL-13 enhance Th2 cell differentiation and stimulate the activation of M2 macrophages, which have anti-inflammatory and regenerative functions. Recent studies indicate that chemotherapeutic agents such as docetaxel, doxorubicin, paclitaxel, and cyclophosphamide can generate damage-associated molecular patterns (DAMPs), which lead to a predominance of Th2 responses and promote the transition to M2 macrophages (Roh et al., 2020; Nurlaila et al., 2022).

In the early stages of lymphedema, there is an increase in M1 macrophages, but differentiation towards M2 macrophages is more pronounced. This shift elevates the expression of VEGF-C and VEGF-A, thereby promoting lymphangiogenesis, and increases iNOS expression, inhibiting contraction of collecting lymphatics (Park et al., 2018; Fu and Liu, 2023). Th1 cells activate macrophages via IFN-γ and modulate chronic inflammation through IL-6. The macrophages also promote lymphangiogenesis by expressing VEGF-C and VEGF-A. Th17 cells secrete IL-17A, which binds to the IL-17R complex, activating NF-κB activator 1/TNF receptor-associated factor 6 (Act1-Traf6) pathway and ultimately leading to the activation of NF-κB signaling. Additionally, Th17 cells inhibit lymphatic vessel formation, resulting in reduced expression of LEC markers such as Prox-1 and LYVE-1 (Park et al., 2018; Fu and Liu, 2023). At this stage, macrophages are the major type of VEGF-C expressing cells (Gousopoulos et al., 2017b). In animal models, it has been revealed that during the initial stages of lymphedema, inflammation involving macrophages contributes to the modulation of hypoxia-inducible factor-1α (HIF-1α). However, it is not necessarily expressed in later stages and can be utilized as a supplemental tool during the initial inflammatory phase. These cells play a role in upregulating the VEGF-C/VEGFR-3 signaling pathway during the early stages of lymphedema. (Ogata et al., 2016; Sung et al., 2022). Although VEGF-C levels increase in lymphedematous tissue, T-cell derived cytokines such as IL-4, IL-13, IFN-γ, and TGF-β1 directly affect LECs, reducing their responsiveness to lymphangiogenic factors. This ultimately inhibits the formation of functional lymphatic vessels and leads to the development of immature and leaky lymphatic vessels, exacerbating lymphatic fluid stasis. These factors highlight the limitations of therapies targeting VEGF-C in treating lymphedema (Shin et al., 2015; Ogata et al., 2016).

In the late stages of lymphedema, where the condition is fully established, macrophage depletion results in reduced VEGF-C expression, increased Th2 cell accumulation, and collagen deposition. Consequently, this leads to an increase in fibrosis and a decrease in lymphatic pumping and collateral lymphatic formation, exacerbating the severity of lymphedema. Additionally, T reg cells increase in number within lymphedematous tissues and help regulate the immune response. They mitigate chronic inflammation by inhibiting various immune cells, including Th1, Th2 cells, macrophages, neutrophils, and dendritic cells, contributing to a homeostatic mechanism that controls disease progression (Duhon et al., 2022; Brown et al., 2023b).

Using a mouse lymphedema model, researchers studied the changes in the types of immune cells present over time. CD45^+^ cells, which play a crucial role in the activation and differentiation of T cells through the T cell receptor, continued to increase for up to 6 weeks. At 2 weeks after surgery, they exhibited twice as many cells compared to normal mice that did not undergo surgery. At the same time, Ly6G^+^ and CD4^+^ cells, representing myeloid and lymphoid cells, respectively, were the predominant cell types and increased compared to pre-surgery levels. CD8^+^ cells, monocytes (Ly6C^+^), and macrophages (CD11c^+^F4/80^+^) also increased during this period, which coincided with the reduction in lymphatic vessel contractility. F4/80^−^CD68^+^ macrophages, and CD206^+^ cells peaked at 4 weeks (Gousopoulos et al., 2016).

In another study, the blockade of leukotriene B4 (LTB4) resulted in a reduction in the infiltration of macrophages, neutrophils, and CD4^+^ T cells. LTB4, a biologically active lipid, is an arachidonic acid metabolite produced by pro-inflammatory immune cells, including dendritic cells, macrophages, eosinophils, mast cells, and neutrophils. Upon binding to its cognate G protein-coupled receptor, LTB4 elicits a potent inflammatory response (Jo-Watanabe et al., 2019). It was observed that LTB4, when binding to the BLT1 receptor, mediated the recruitment of CD4^+^ and CD8^+^ T cells to inflammatory tissues and promoted the differentiation of Th17 cells. This indicated that LTB4 is involved in both innate immunity and T cell responses (Jiang et al., 2018). LTB4 acts as a strong chemoattractant and leukocyte activator, particularly exerting its role as one of the most potent lipid chemotactic factors for neutrophils. The recruitment of monocytes and macrophages mediated by LTB4 is associated with chronic diseases such as obesity, insulin resistance, and type 2 diabetes (Jiang et al., 2018). Recent studies have revealed that LTB4 elevation contributes to increased insulin resistance in obese mice, raising the speculation that it may also impact adipose deposition in secondary lymphedema following surgery (Murtomaki et al., 2014). Moreover, at a low concentration of 10 nM, LTB4 exhibits a pro-lymphangiogenic effect. In contrast, at a higher concentration of 400 nM, it inhibits VEGFR3 mRNA expression and VEGFR3 protein phosphorylation while also interfering with Notch signaling, thereby hindering the development and maintenance of lymphatic vessels (Jiang et al., 2018). A study in a mouse model revealed that NSAIDs like ketoprofen effectively diminish inflammation by inhibiting 5-lipoxygenase (5-LO), the enzyme responsible for LTB4 synthesis. This inhibition also leads to the induction of pro-lymphangiogenic factors (Nakamura et al., 2009; Bertelli et al., 2020).

As a mechanism of lymphedema, inflammation is involved, but from a different perspective, one can also consider the changes in lymphatic vessels that occur during inflammatory responses. In one study, acute inflammatory reactions and chronic inflammatory diseases such as psoriasis, atopic dermatitis, and inflammatory bowel disease were associated with lymphangiogenesis in lymphatic vessels and the hyperplasia and expansion of pre-existing lymphatic vessels, resulting in an increase in lymphatic vascular density (Kunstfeld et al., 2004). Inflammatory responses involve the infiltration of CD11^+^/Gr-1^+^ macrophages, leading to the upregulation of VEGF-C, VEGF-D, and VEGF-A expression, which play a role in antigen clearance and inflammation resolution (Kataru et al., 2009). VEGF-C binds to VEGFR-3 and NRP-2, and through proteolytic cleavage, it also binds to VEGFR2, thereby inducing inflammatory lymphangiogenesis (Tammela and Alitalo, 2010). Furthermore, in inflammatory lymphatic vessel expansion, VEGF-A acts as a major inducer and is highly expressed in inflammatory diseases (Nagy et al., 2002). Through the upregulation of VEGF-C and VEGF-D expression in skin inflammatory responses, it has been demonstrated that lymphatic vessel activation, subsequent lymphatic expansion, fluid drainage, and anti-inflammatory effects occur in both acute and chronic inflammation (Dieterich et al., 2014).

### Pathophysiology of secondary lymphedema: adipose expansion and remodeling

Adipose tissue deposition is a pathological feature observed in the late stages of lymphedema, and it has attracted significant attention from researchers in recent years. Some studies have reported that lymphatic fluid stasis promotes adipose differentiation (Azhar et al., 2020; Li et al., 2020). In response to lymphatic fluid stasis, there is an increased expression of CCAAT/enhancer-binding protein α (C/EBP-α) and peroxisome proliferator-activated receptor-γ (PPAR- γ) (Aschen et al., 2012; Koc et al., 2021; Sung et al., 2022; Hsiao et al., 2023). These factors are known to be key regulators of adipogenesis, which includes the differentiation, proliferation of adipocytes and lipid accumulation. In particular, macrophages respond to lymphatic fluid stasis by inducing the expression of PPAR-γ, which contributes to the generation of inflammatory cytokines and adipose tissue inflammation. PPAR-γ is also expressed by other cell types such as adipocytes, pericytes, and LECs (Aschen et al., 2012). C/EBP-α is primarily required for the activation of PPAR-γ, and continuous PPAR-γ expression is essential for adipocyte differentiation and lipid accumulation, making it the principal transcription factor in adipogenesis (Rosen, 2002; Gesta et al., 2007). Furthermore, C/EBP-α-mediated PPAR-γ expression is known to act as a positive feedback loop, further promoting adipogenesis (Cook and Cowan, 2008).

Accumulated lymph contains higher levels of insulin and insulin-like growth factor-2 (IGF-2), which promote the expression of adipogenesis genes such as C/EBP-α, PPAR-γ, and fatty acid-binding protein 4 (FABP4), inducing adipogenesis and differentiation of adipose-derived stem cells (ASCs) into adipocytes. Furthermore, this process leads to the accumulation of adipose tissue, resulting in increased secretion of adipokines such as adiponectin and resistin. This, in turn, leads to increased secretion of insulin, contributing to the vicious cycle of lymphedema (Hsiao et al., 2023).

In response to lymphatic fluid stasis, adiponectin expression increases, and it is expressed not only by macrophages but also by adipocytes and fibroblasts. Adiponectin acts as a peptide hormone and serves as a late marker of activated adipocytes, with its expression remaining high during periods of lipid accumulation. However, its expression decreases in cases of adipose tissue hypertrophy or hypoxia (Harford et al., 2011; Aschen et al., 2012). It plays a dual role in the context of lymphatic obstruction. In the early stages, it initiates an inflammatory response through macrophage activation. Conversely, in the later stages, it has a dual effect by reducing monocyte adhesion, thereby inhibiting macrophage activation (Aschen et al., 2012).

**FIGURE 2:**
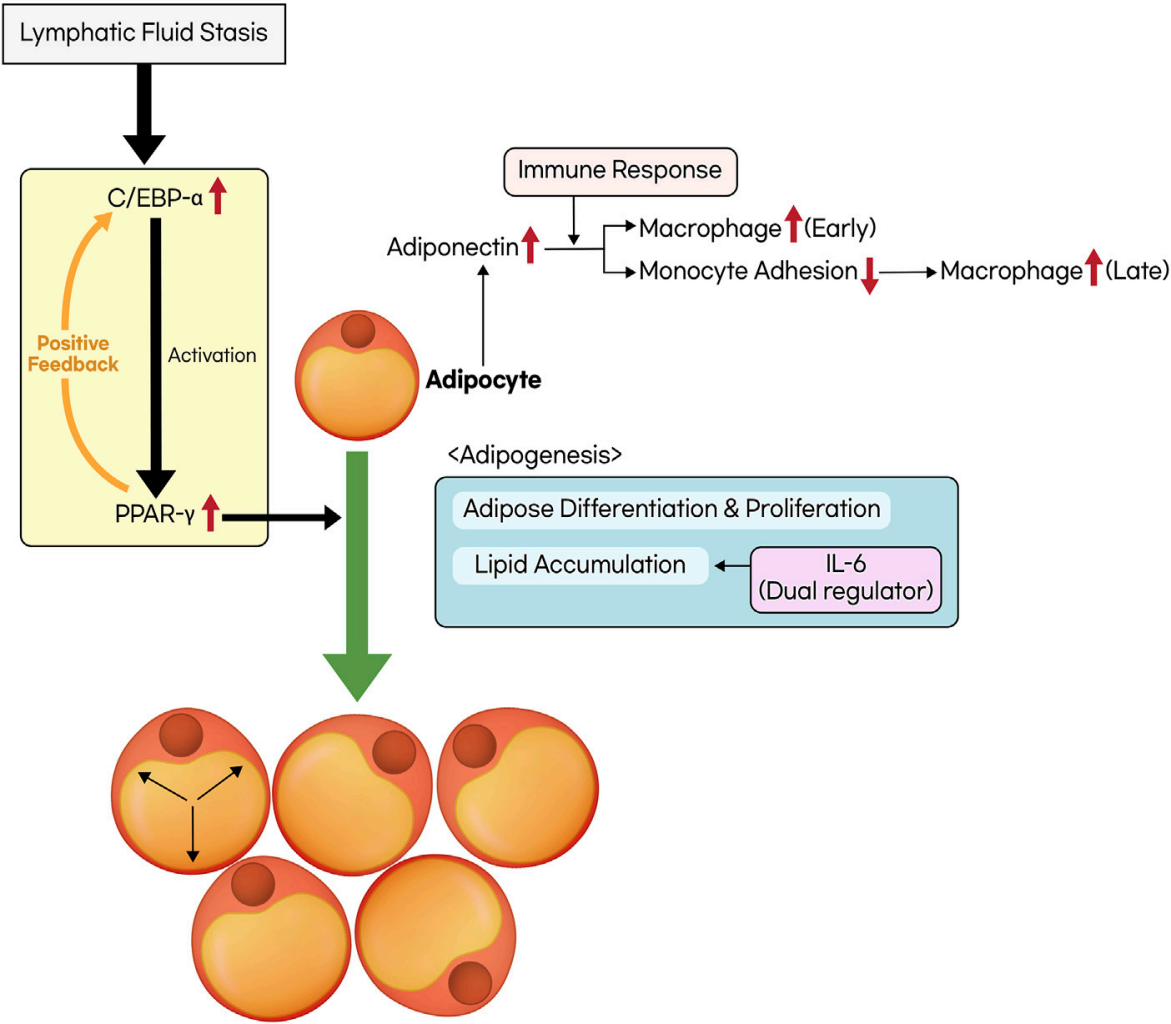
The process of adipose tissue deposition and remodeling. Lymphatic fluid stasis leads to an upregulation in the expression of CCAAT/enhancer-binding protein α (C/EBP-α) and peroxisome proliferator-activated receptor-γ (PPAR-γ). C/EBP-α is a prerequisite for the activation of PPAR-γ, and they mutually establish a positive feedback loop. The activation of PPAR-γ facilitates adipogenesis, which includes processes such as adipose differentiation, proliferation, and lipid accumulation. Interleukin-6 (IL-6) possesses a dual role in regulating lipid accumulation. In addition, the activation of adipocytes triggers the secretion of adiponectin, which exhibits distinct effects on inflammatory responses at both the early and late stages.

Prox-1 serves as the master regulator of lymphatic development. Prox-1 knockout mice developed chylothorax, and its inactivation led to obesity (Sosa-Pineda et al., 2000; Escobedo et al., 2016). Haploinsufficiency of the Prox-1 gene caused adult-onset obesity because of abnormal lymph leakage from irregularly patterned and ruptured lymphatic vessels (Harvey et al., 2005). Recent studies have shown that fatty acids from lymphatic fluid directly contribute to adipocyte proliferation and differentiation. This corresponds to the increased expression of adipogenic markers like adiponectin and C/EBP-α in a mouse lymphedema model (Aschen et al., 2012; Cuzzone et al., 2014). Furthermore, the proinflammatory cytokine IL-6 acts as both a negative and positive regulator of adipose deposition, playing a homeostatic role in limiting the extent of adipose accumulation (Avraham et al., 2013). Considering the effects of CD4^+^ T cell deficiency and Th2 differentiation inhibition in reducing adipose deposition, the extent of adipose deposition is closely related to the severity of lymphatic dysfunction and inflammation (Li et al., 2015) (Figure 2.).

Obesity can impact the structure of lymphatic vessels even in the absence of lymphatic injury. In mice models, exposure of LECs to free fatty acids (FFAs) increases the expression of apoptosis-related genes such as caspase-3 and Annexin V. Additionally, in obese mice, there is downregulation of Prox-1, VEGFR-3, and chemokine ligand 21 (CCL21) expression, along with upregulation of the pro-apoptotic gene Bcl-2 associated X protein (Bax) and the inflammatory cell receptor intercellular adhesion molecule-1 (ICAM-1). This leads to increased vulnerability of LECs to apoptosis, as APCs become trapped in peripheral tissues (García Nores et al., 2016; Khan et al., 2022). In obesity, diet-induced effects compromise the lymphatic system by disrupting lymphatic transport and lymph node structure, as well as dendritic cell mobility. This is partly due to obesity-induced inflammation from T and B cells (Weitman et al., 2013). Moreover, an increase in nitric oxide production within the perilymphatic tissues, driven by macrophages and smooth muscle cells producing iNOS under the influence of prostaglandin E2 (PGE2), causes lymphatic vessels to dilate and reduces their pumping capacity, which can further contribute to the development of lymphedema (Torrisi et al., 2016).

A high-fat diet without obesity (HFD) does not exacerbate lymphedema, as it has been elucidated that the impairment of lymphatic function is determined by adiposity rather than the content of the diet (Gousopoulos et al., 2017a). Furthermore, recent research utilizing a mouse tail lymphedema model has demonstrated that a HFD increases serum β-hydroxybutyrate (β-OHB) levels, leading to the induction of VEGF-C and subsequently increasing lymphangiogenesis. Given that VLNT involves lymphangiogenic mediators such as VEGF-C, the study suggests that combining VLNT with HFD may enhance the effectiveness of the surgery (Choi et al., 2023).

In a study on obesity-associated lymphedema, exposure of LECs to FFAs was treated with agents targeting intracellular signaling pathways, including PTEN inhibitor (PTENi) inhibiting the conversion of phosphatidylinositol-3,4,5-trisphosphate (PIP3) to phosphatidylinositol-4,5-bisphosphate (PIP2), recombinant VEGF-C, and insulin indirectly activating PIP3. The results of culture experiments showed normalization of the expression levels of VEGFR-3, p-AKT, p-eNOS, and Prox-1 (Khan et al., 2022).

### Pathophysiology of secondary lymphedema: tissue fibrosis

The activation of CD4^+^ cells due to lymphatic vessel damage promotes differentiation into Th2 cells more than Th1 cells. Consequently, this leads to the induction of profibrotic cytokines and growth factors such as IL-4, IL-13, and TGF-β1, resulting in tissue fibrosis and leaky lymphatics. This, in turn, reduces lymphatic pumping and collateral lymphatic formation (Savetsky et al., 2014). In the progression of lymphedema, the inflammatory response is closely linked to an increase in iNOS, which elevates nitric oxide (NO) levels. This elevation inhibits lymphatic contraction, crucial for the lymphatic system’s function. As lymphedema advances, smooth muscle cells (SMCs) in the lymphatic vessels progressively lose their contractile function. This loss is due to a phenotypic shift from a contractile to a synthetic form, reducing their ability to contract and contributing instead to collagen fiber synthesis. This process leads to the remodeling of surrounding tissues and, ultimately, to the progressive fibrosis of collecting lymphatic vessels in the end-stage of the disease. Such fibrosis results in the replacement of normal lymphatic tissue with scar tissue, a process known as lymphangiosclerosis, which narrows the lumen of the lymphatic vessels and can lead to end organ failure (Ogata et al., 2015; Sung et al., 2022).

Blocking the differentiation of Th2 cells using IL-4 and IL-13 antibodies resulted in reduced lymphatic fibrosis and improved lymphatic function. (Avraham et al., 2013). Furthermore, *in vitro* and *in vivo* studies have demonstrated that IL-4 and IL-13 downregulate the LEC-specific transcription factor Prox-1 and the LEC marker LYVE-1, adversely affecting LEC survival, proliferation, and tubule migration (Savetsky et al., 2015). In one study, hyaluronidase was shown to increase the activity of Th1 cells and reduce the activity of Th2 cells in a hindlimb postsurgical lymphedema model, thus reversing tissue fibrosis. This finding confirmed the significant role of Th2 cells in fibrosis (Cho et al., 2017).

**FIGURE 3:**
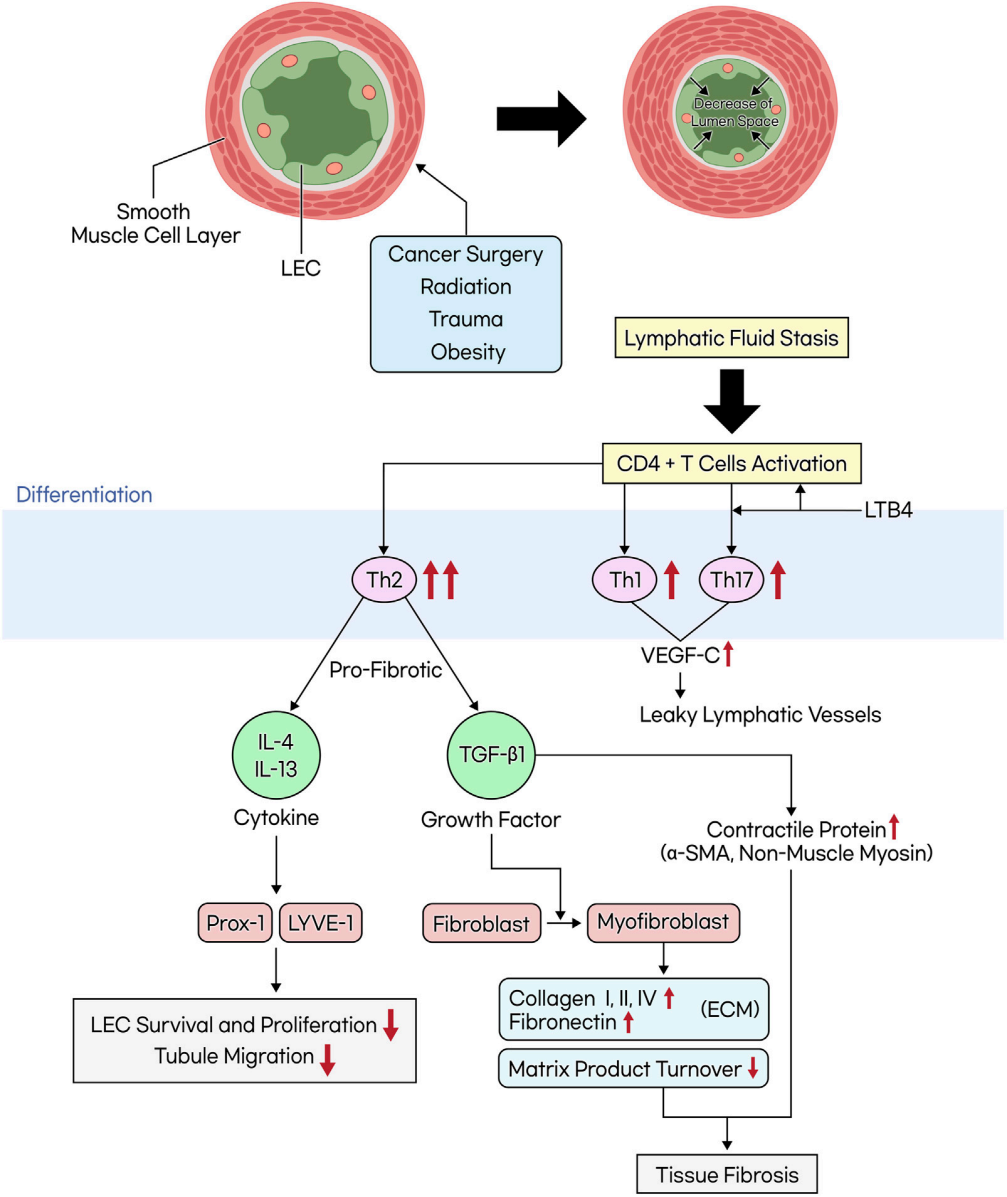
The mechanism of tissue fibrosis occurring in lymphedema. When lymphatic vessels are damaged due to cancer surgery, radiation, trauma, or obesity, the smooth muscle cells within these vessels thicken and transform. This transformation leads to the narrowing of the lymphatic lumen. Additionally, an increase in inner pressure within the lymphatic vessels causes the junctions between lymphatic endothelial cells to weaken, which in turn leads to increased lymph leakage and exacerbates lymphatic fluid stasis. This stasis activates CD4^+^ T cells, favoring differentiation into Th2 cells over Th1 cells. Th2 cells secrete profibrotic cytokines (IL-4, IL-13) and growth factors (TGF-β1). TGF-β1 induces the differentiation of fibroblasts into myofibroblasts, promoting the accumulation of the extracellular matrix (ECM) and the production of contractile proteins, while reducing matrix product turnover, ultimately leading to tissue fibrosis.

TGF-β1 is a profibrotic and anti-lymphangiogenic growth factor that, when activated, promotes the differentiation of fibroblasts into myofibroblasts. In this process, the formation of gap junctions and the expression of contractile proteins such as α-SMA and non-muscle myosin result in a contractile phenotype, making it a cellular effector of fibrosis. Originally, myofibroblasts play a role in tissue repair during wound healing. However, as mentioned earlier, in pathological conditions like lymphedema, they are associated with fibrosis. As a result, it increases collagen production and reduces matrix product turnover, promoting the deposition of ECM components such as fibronectin, collagen types I, II, and IV. In particular, collagen type III has been found to exhibit a 39-fold higher gene transcription level compared to collagen type I in stage III lymphedema. Further research targeting this aspect is warranted (Biernacka et al., 2011; Karayi et al., 2020; Nurlaila et al., 2022) (Figure 3.). TGF-β1 forms a complex with fibroblasts by binding to type II and III receptors, subsequently phosphorylating the downstream effector, Smad. This activation of the TGF-β1/Smad pathway enhances the production of ECM components and inhibits fibroblast expression of matrix metalloproteinase-1 (MMP-1). This inhibition prevents the degradation of collagen fibers within the matrix, a crucial process in tissue remodeling and fibrosis (Yuan and Varga, 2001; Meng et al., 2016).

In skin biopsies from unilateral breast cancer-related lymphedema patients, increased expression of TGF-β1, CD26^+^ fibroblasts, and ECM molecules was observed. In mouse models, inhibition of TGF-β1 led to a reduction in ECM deposition, an increase in collateral lymphatics, and a suppression of T-cell infiltration. There have been studies in mouse models using a TGF-β1 receptor kinase inhibitor, which reduced the severity of lymphedema and increased lymphangiogenesis. Additionally, Pirfenidone, an FDA-approved drug for inhibiting TGF-β1 activity, originally used for the treatment of pulmonary fibrosis, showed a reduction in mRNA expression of TGF-β1 signaling molecules, fibrotic genes, decreased staining of type 1 collagen, and a decrease in the number of pSmad3+ cells when topically applied to mouse models. Furthermore, it was found to inhibit the infiltration of CD4^+^ T cells, Th1 cells, and Th2 cells, excluding macrophages (Yoon et al., 2020; Baik et al., 2022).

During embryogenesis, exposing LEC progenitors emerging from the cardinal vein to the soft matrix of embryonic tissue increases the expression of the transcription factor GATA2, facilitating cellular migration and enhancing sensitivity to VEGF-C. It has recently been discovered that LECs, similar to BECs, are sensitive to the degree of matrix stiffness. This suggests that when fibrosis occurs due to TGF-β1, an increase in ECM stiffness may hinder the proper binding and network formation between cells, thereby impairing lymphatic function (Frye et al., 2018; Baik et al., 2022).

### Pharmacologic treatment of secondary lymphedema

The pathophysiology of lymphedema is intricate, rendering lymphangiogenesis promotion alone inefficient. Therapeutic strategies are diversified into lymphangiogenic interventions using cytokines, anti-inflammatory treatments, and anti-fibrotic therapies, all of which are under both preclinical and clinical investigation (Brown et al., 2022). Lymphangiogenic growth factors like VEGF-C and VEGF-D activate VEGFR-3 and have been applied in various forms such as recombinant human VEGF-C, virus-mediated gene therapy, topical formulations, integration with nanofibrillar collagen scaffolds, combinations with adipose-derived stem cells, or as VEGF-C mRNA lipid nanoparticle injections to enhance functional lymphatic vessel formation (Karkkainen et al., 2001; Hwang et al., 2011; Kim et al., 2013; Nguyen et al., 2021; Nguyen et al., 2022). A combined treatment involving VLNT and Lymfactin^®^ (adenoviral type 5-based gene therapy vector that expresses human VEGF-C) has undergone phase I and II clinical trials (Hartiala et al., 2020). However, the efficacy of VEGF-C as a standalone treatment is questionable due to its increased levels in lymphedema and its balance with anti-lymphangiogenic cytokines (Jensen et al., 2015; Brown et al., 2022). Additionally, agents like fibroblast growth factor 2 (FGF2), Hepatocyte growth factor (HGF), and retinoic acid agonists like 9-cis retinoic acid have been identified to promote lymphangiogenesis through their respective pathways (Javerzat et al., 2002; Kajiya et al., 2005; Onishi et al., 2014; Daneshgaran et al., 2020).

Anti-inflammatory approaches include drugs like Ketoprofen, an NSAID that inhibits the leukotriene B4 pathway, and Bestatin, a selective antagonist, both showing potential in animal models and ongoing clinical trial (Tian et al., 2017; Rockson et al., 2018). Tacrolimus works by inhibiting nuclear factor of activated T-cells (NFAT) signaling, reducing IL-2 expression and exerting an immunosuppressive effect on CD4^+^ T cells; when used topically, it has been shown to enhance lymphatic function (Clipstone and Crabtree, 1992; Chow et al., 1999; Liao et al., 2013; Gardenier et al., 2017). The role of Th2 differentiation in lymphedema pathophysiology suggests that using neutralizing antibodies against IL-4 and IL-13 can reduce inflammation and improve lymphatic function (Avraham et al., 2013; Mehrara et al., 2021). Doxycycline has shown effectiveness particularly in filariasis-induced lymphedema by inhibiting Th2 differentiation, monocyte recruitment, and macrophage polarization (Furlong-Silva et al., 2021). Lastly, the contribution of TGF-β1 to lymphedema fibrosis can potentially be mitigated with small molecule inhibitors or neutralizing antibodies, which have been shown to reduce the severity of lymphedema and enhance collateral lymphatic formation (Sano et al., 2020; Yoon et al., 2020).

### Conservative and surgical treatment of secondary lymphedema

The treatment approach for lymphedema varies based on the severity of the condition, with the International Society of Lymphology (ISL) staging system commonly employed (International Society of Lymphology, 2013). This staging system categorizes lymphedema based on the dominance of lymphatic fluid and fibroadipose tissue. Stage 0, known as subclinical lymphedema, is characterized by the patient experiencing symptoms without visible edema. Stage I presents as reversible limb swelling and pitting edema, indicating fluid predominance. Stage II marks the transition to Irreversible limb swelling, signifying the onset of fibroadipose tissue dominance, and hence, the absence of pitting edema. Stage III is the end-stage of lymphedema, exhibiting severe swelling, trophic skin changes, and elephantiasis. Consequently, when considering surgical interventions, it is essential to take into account the dominance of fluid and fibroadipose tissue. According to one study, for up to stage I, lymphaticovenular anastomosis (LVA) is suggested, while for stage I and beyond, vascularized lymph node transfer (VLNT) can be considered. For stage II and higher, additional methods like liposuction and excision may be viable options (Lin et al., 2022).

In the treatment of lymphedema, non-surgical interventions such as compression, massage, skin care, and exercise are prioritized (Lurie et al., 2022). Complete decongestive therapy (CDT), a multimodal approach, plays a central role as the primary non-surgical treatment for lymphedema and should be individualized based on the patient’s severity and condition (Senger et al., 2023). Treatment progresses to the maintenance phase, Phase II, once Phase I is completed, aiming for maximal volume reduction and improved skin texture. Phase I focuses on educating patients about lymphedema, particularly as an inflammatory condition leading to interstitial fibrosis, subcutaneous fibrous tissue formation, and subcutaneous fat deposition. Phase I also includes skin and nail care, manual lymph drainage (MLD) and 24-h multilayered low-stretch bandaging (MLB) to enhance lymph collector transport capacity (Michopoulos et al., 2020). Phase II includes compression garments and lymphedema exercises. When compression garments were worn for over 24 weeks following MLB, there was a significant improvement, with a volume reduction of 31% observed after 24 weeks. In contrast, when compression garments were used alone without prior MLB, the volume reduction was approximately halved, reaching only 15.5% after 24 weeks (Badger et al., 2000). Complementary therapies such as low-level laser therapy (LLLT), elastic taping, ultrasound, and acute puncture are available, but according to the Putting Evidence into Practice guidelines, only LLLT and elastic band therapy have been classified as ‘likely to be effective’ (Rodrick et al., 2014).

In cases where conservative treatment is ineffective for lymphedema, surgical intervention is considered (Allen Jr. and Cheng, 2016; Masià et al., 2016; Schaverien and Coroneos, 2019b). Surgical treatment options can be categorized into excisional treatment, which includes procedures such as liposuction and direct excision with skin grafting (such as the Charles procedure, Sistrunk operation, and Thompson’s operation), and physiological treatment, which encompasses flap interposition and lymphatic bypass. Physiological treatment involves methods for restoration of the lymphatic drainage. It is divided into flap interposition and lymphatic bypass. Lymphatic bypass includes lymphatic-lymphatic bypass, lymphovenous bypass (LVB), LVA, and VLNT. Excisional treatment is considered in severe cases of lymphedema, including those with recurrent infections, skin ulcers, chronic pain, and a substantially diminished quality of life due to its significant morbidity. In recent times, combinations of excisional and physiologic treatment have been proven to have better results compared to stand-alone procedures (Schaverien and Coroneos, 2019a; Viviano and Neligan, 2022).

Lymphaticovenular anastomosis (LVA) is a super-microsurgery technique that involves anastomosis between lymphatic vessels and venules to drain stagnant lymphatic fluid in lymphedema patients (O'Brien et al., 1977; Poumellec et al., 2017). This procedure connects lymphatic vessels smaller than 0.8 mm in diameter to corresponding venules and has gained international recognition as an effective surgical treatment for lymphedema. The process includes making incisions on the affected limb, identifying lymphatic channels and suitable veins with dyes or indocyanine green (ICG), and ensuring the veins demonstrate no backflow. Various anastomotic techniques, including end-to-end, end-to-side, and side-to-end, are employed, with the choice of technique depending on the specific case requirements (Gallagher et al., 2020). The patency of the anastomoses is typically confirmed with intra-operative ICG, but the optimal number of anastomoses remains under debate (Schaverien and Coroneos, 2019b). While there is no standardized method for comparing the results of LVA and LVB, both approaches have shown long-term volume reduction, with a reduction of 73% in 75% of patients and 44% in 42% of patients, respectively. Furthermore, a significant reduction in cellulitis has also been observed with both methods (O'Brien et al., 1990; Campisi et al., 2010; Chang, 2012; Cormier et al., 2012; Yamamoto and Koshima, 2014).

VLNT represents the most recent and advanced method. While the indications are not yet well-defined, it may be considered in cases of total occlusion observed in lymphoscintigraphy, recurring cellulitis in ISL stage II, the absence of acute cellulitis, and no improvement even after 6 months of CDT (Poon and Wei, 2014). It is a sophisticated microsurgical method that relocates lymph nodes along with their blood vessels from one part of the body to another to enhance lymphatic drainage in limbs that have impaired function. It is believed to facilitate the regeneration of lymphatic drainage primarily by stimulating the growth of new lymphatic vessels through the secretion of growth factors, such as vascular endothelial growth factor (VEGF) (Aschen et al., 2014; Suami et al., 2016) and by acting as a “pump” that aids in redirecting the lymphatic fluid back into the circulation, thus improving lymphatic system function in the affected limbs (Cheng et al., 2014). Vascularized lymph nodes are harvested from regions such as the groin, thoracic, submental, and supraclavicular areas and then transplanted to the upper extremities—specifically the wrist, elbow, and axillary regions—or to the lower extremities, including the ankle and groin, using a free transfer technique (Gallagher et al., 2020). It is well-recognized that preserving vascular supply during the transfer process significantly influences the extent of improvement in lymphedema and the enhancement of lymphatic vessel function (Tobbia et al., 2009). Although the vessels are very small, flap elevation requires a highly precise technique using the free-style free flap method, and studies on VLNT are still in their early stages, a reported volume reduction of 47% has been documented (Cormier et al., 2012; Michopoulos et al., 2020). Generally considered safe, VLNT may present complications such as flap loss, donor site lymphedema, seroma, lymphocele, infection, and more. Nonetheless, VLNT has introduced new possibilities for physiological treatment in advanced-stage lymphedema (Michopoulos et al., 2020).

## Conclusion

While existing surgical and conservative treatments aim to restore lymphatic function, they often do not fully address the damage already done to lymphatic vessels. Recent molecular research has shed light on potential gene and protein therapies by focusing on the signaling pathways critical to lymphedema’s pathophysiology. Although most current research is in preliminary stages, using *in vitro* or animal models, these studies pave the way for future applications in humans and the development of effective drug therapies. Currently, there are no FDA-approved drug treatments for lymphedema, however ongoing research holds promise. Understanding the complex interactions of inflammatory responses that drive the pathogenesis of lymphedema is essential. Further dissecting the cellular and molecular aspects of this condition will help refine existing treatments and foster the creation of innovative therapeutic strategies. Future research should not only deepen our comprehension of these mechanisms but also include clinical trials to evaluate new treatments’ effectiveness and safety in human subjects. Given the diverse clinical presentations and the complex nature of lymphedema, personalized treatment strategies are likely necessary for effective management. Integrating detailed molecular insights with clinical practice is crucial for developing tailored approaches that optimize patient outcomes.
